# A new pooling strategy for high-throughput screening: the Shifted Transversal Design

**DOI:** 10.1186/1471-2105-7-28

**Published:** 2006-01-19

**Authors:** Nicolas Thierry-Mieg

**Affiliations:** 1 Laboratoire Logiciels-Systèmes-Réseaux, IMAG Institute, BP53, 38041 Grenoble Cedex 9, France

## Abstract

**Background:**

In binary high-throughput screening projects where the goal is the identification of low-frequency events, beyond the obvious issue of efficiency, false positives and false negatives are a major concern. Pooling constitutes a natural solution: it reduces the number of tests, while providing critical duplication of the individual experiments, thereby correcting for experimental noise. The main difficulty consists in designing the pools in a manner that is both efficient and robust: few pools should be necessary to correct the errors and identify the positives, yet the experiment should not be too vulnerable to biological shakiness. For example, some information should still be obtained even if there are slightly more positives or errors than expected. This is known as the group testing problem, or pooling problem.

**Results:**

In this paper, we present a new non-adaptive combinatorial pooling design: the "shifted transversal design" (STD). It relies on arithmetics, and rests on two intuitive ideas: minimizing the co-occurrence of objects, and constructing pools of constant-sized intersections. We prove that it allows unambiguous decoding of noisy experimental observations. This design is highly flexible, and can be tailored to function robustly in a wide range of experimental settings (i.e., numbers of objects, fractions of positives, and expected error-rates). Furthermore, we show that our design compares favorably, in terms of efficiency, to the previously described non-adaptive combinatorial pooling designs.

**Conclusion:**

This method is currently being validated by field-testing in the context of yeast-two-hybrid interactome mapping, in collaboration with Marc Vidal's lab at the Dana Farber Cancer Institute. Many similar projects could benefit from using the Shifted Transversal Design.

## Background

With the availability of complete genome sequences, biology has entered a new era. Relying on the sequencing data of genomes, transcriptomes or proteomes, scientists have been developing high-throughput screening assays and undertaking a variety of large scale functional genomics projects. While some projects involve quantitative measurements, others consist in applying a basic yes-or-no test to a large collection of samples or "objects", – be they individuals, clones, cells, drugs, nucleic acid fragments, proteins, peptides... A large class of these binary tests aims at identifying relatively rare events. The main goal is of course to obtain information as efficiently and as reliably as possible. Typically, this is achieved by minimizing the cost of the basic assay in terms of time and money, and automating and parallelizing the experiments as much as possible. A major difficulty stems from the fact that high-throughput biological assays are usually somewhat noisy: reproducibility is a known problem of microarray analyses, and both false positive and false negative observations are to be expected in binary type experiments. These experimental artifacts should be identified and properly treated. A clean way to deal with the issue consists in repeating all tests several times, but this is usually prohibitively expensive and time-consuming. A more practical approach, in the case of binary tests, consists in retesting all positive results obtained in a first round. This strategy identifies most of the false positives at a reduced cost, but is powerless with regard to false negatives, leaving us in need of a better solution.

In the case of binary experiments testing for rare events, an intuitively appealing strategy consists in pooling the samples to minimize the number of tests. It requires three conditions. First, the objects under scrutiny must be available individually, in a tagged form. For example, a cDNA library in bulk is not exploitable, but a collection of cDNA clones or of cloned coding regions, such as the one produced by the *C. elegans *ORFeome project [1], is fine. Second, it must be possible to test a pool of objects in a single assay and obtain a positive readout if at least one of the objects is positive. For example, this is the case when searching for a specific DNA sequence by PCR in a mixture of molecules: a product will be amplified if at least one of the pooled molecules contains the target sequence. Third, pooling is especially desirable and efficient when the fraction of expected positives is small (at most a few percent). Under these conditions, pooling strategies can be applied, and the difficulty then consists in choosing a "good" set of pools. This being an intuitive but rather vague goal, it must be formalized. A simple formulation, known as the group testing problem (or pooling problem), is the following. Consider a set of n events which can be true or false, represented by n Boolean variables. Let us call "pool" a subset of variables. We define the value of a pool as the disjunction (i.e., the logical OR operator) of the variables that it contains. Let us assume that at most t variables are true. The goal is to build a set of v pools, where v is small compared to n, such that by testing the values of the v pools, one can unambiguously determine the values of the n variables.

If the pools must be specified in a single step, rather than incrementally by building on the results of previous tests, the problem is called "non-adaptive". Although adaptive designs can require fewer tests, non-adaptive pooling designs are often better suited to high-throughput screening projects because they allow parallelization and facilitate automation of the experiments, and also because the same pools can be used for all targets, thereby reducing the total project cost.

The ability to deal with noisy observations is an important added benefit to using a pooling system, compared to the classical individual testing strategy. Indeed, noise detection and correction capabilities are inherent in any pooling system, because each variable is present in several pools, hence tested many times. Depending on the expected noise level, the redundancy can be chosen at will, and simply testing a few more pools than would be necessary in the absence of noise results in robust error-correction. It should be noted that minimization of the number of pools and noise correction are two conflicting goals: increasing noise tolerance generally requires testing more pools. Designing a set of pools requires balancing these two objectives, and finding the right compromise to suit the application.

Other application-dependent constraints may be imposed. In particular, the pool sizes are often limited by the experimental setting. For example, in the context of the *C. elegans *protein interaction mapping project led by Marc Vidal [2,3], it is estimated that, using their high-throughput two-hybrid protocol, reliable readouts can be obtained with pools containing 400 AD-Y clones, or perhaps up to 1000 by tweaking the assay (Marc Vidal, personal communication).

Many groups have used with some success variants of the simple "grid" design, which consists in arraying the objects on a grid and pooling the rows and columns [e.g. [4-6]]. However, although it is better than no pooling, this rudimentary design is vulnerable to noise and behaves poorly when several objects are positive, in addition to being far from optimal in terms of numbers of tests.

In answer to its shortcomings, more sophisticated error-correcting pooling designs have been proposed. Some of these designs are very efficient in terms of numbers of tests, but lack the robustness and flexibility that most real biological applications require. Others are more adaptable and noise-tolerant at the expense of performance. In this paper, we present a new pooling algorithm: the "shifted transversal design" (STD). This design is highly flexible: it can be tailored to allow the identification of any number of positive objects and to deal with important noise levels. Yet it is extremely efficient in terms of number of tests, and we show that it compares favorably to the previously described pooling designs.

The paper is organized as follows. After providing a formal definition STD, we show that it constitutes an error-correcting solution to the pooling problem. The theoretical performance of STD is then evaluated and compared with the main previously described deterministic pooling designs. Finally, we summarize our results and discuss future directions.

## Results (1): the Shifted Transversal Design

### Preliminaries

The following notations are used throughout this paper, in accordance with the notations from [7].

Let n ≥ 2, and consider the set An
 MathType@MTEF@5@5@+=feaafiart1ev1aaatCvAUfKttLearuWrP9MDH5MBPbIqV92AaeXatLxBI9gBamrtHrhAL1wy0L2yHvtyaeHbnfgDOvwBHrxAJfwnaebbnrfifHhDYfgasaacH8akY=wiFfYdH8Gipec8Eeeu0xXdbba9frFj0=OqFfea0dXdd9vqai=hGuQ8kuc9pgc9s8qqaq=dirpe0xb9q8qiLsFr0=vr0=vr0dc8meaabaqaciaacaGaaeqabaWaaeGaeaaakeaaimaacqWFaeFqdaWgaaWcbaWexLMBbXgBcf2CPn2qVrwzqf2zLnharCGvLjhzH5wyaGqbaiaa+5gaaeqaaaaa@43C8@ = {A_0_,...,A_n-1_} of n Boolean variables.

We will call "pool" a subset of An
 MathType@MTEF@5@5@+=feaafiart1ev1aaatCvAUfKttLearuWrP9MDH5MBPbIqV92AaeXatLxBI9gBamrtHrhAL1wy0L2yHvtyaeHbnfgDOvwBHrxAJfwnaebbnrfifHhDYfgasaacH8akY=wiFfYdH8Gipec8Eeeu0xXdbba9frFj0=OqFfea0dXdd9vqai=hGuQ8kuc9pgc9s8qqaq=dirpe0xb9q8qiLsFr0=vr0=vr0dc8meaabaqaciaacaGaaeqabaWaaeGaeaaakeaaimaacqWFaeFqdaWgaaWcbaWexLMBbXgBcf2CPn2qVrwzqf2zLnharCGvLjhzH5wyaGqbaiaa+5gaaeqaaaaa@43C8@. We say that a pool is "true", or "positive", if at least one of its elements is true.

Let us call "layer" a partition of An
 MathType@MTEF@5@5@+=feaafiart1ev1aaatCvAUfKttLearuWrP9MDH5MBPbIqV92AaeXatLxBI9gBamrtHrhAL1wy0L2yHvtyaeHbnfgDOvwBHrxAJfwnaebbnrfifHhDYfgasaacH8akY=wiFfYdH8Gipec8Eeeu0xXdbba9frFj0=OqFfea0dXdd9vqai=hGuQ8kuc9pgc9s8qqaq=dirpe0xb9q8qiLsFr0=vr0=vr0dc8meaabaqaciaacaGaaeqabaWaaeGaeaaakeaaimaacqWFaeFqdaWgaaWcbaWexLMBbXgBcf2CPn2qVrwzqf2zLnharCGvLjhzH5wyaGqbaiaa+5gaaeqaaaaa@43C8@.

Let q be a prime number, with q < n.

We define the "compression power" of q relative to n, noted Γ(q,n), as the smallest integer γ such that q^*γ*+1 ^≥ n. We will simply write Γ for Γ(q,n) whenever possible.

Let *σ*_q _be the mapping of {0,1}^q ^onto itself defined by:

∀(x1,…,xq)∈{0,1}q,σq[x1x2⋮xq]=[xqx1⋮xq−1].
 MathType@MTEF@5@5@+=feaafiart1ev1aaatCvAUfKttLearuWrP9MDH5MBPbIqV92AaeXatLxBI9gBaebbnrfifHhDYfgasaacH8akY=wiFfYdH8Gipec8Eeeu0xXdbba9frFj0=OqFfea0dXdd9vqai=hGuQ8kuc9pgc9s8qqaq=dirpe0xb9q8qiLsFr0=vr0=vr0dc8meaabaqaciaacaGaaeqabaqabeGadaaakeaafaqabeqacaaabaGaeyiaIiIaeiikaGIaemiEaG3aaSbaaSqaaiabigdaXaqabaGccqGGSaalcqWIMaYscqGGSaalcqWG4baEdaWgaaWcbaGaemyCaehabeaakiabcMcaPiabgIGiolabcUha7jabicdaWiabcYcaSiabigdaXiabc2ha9naaCaaaleqabaGaemyCaehaaOGaeiilaWcabaacciGae83Wdm3aaSbaaSqaaGqaciab+fhaXbqabaaaaOWaamWaaeaafaqabeabbaaaaeaacqWG4baEdaWgaaWcbaGaeGymaedabeaaaOqaaiabdIha4naaBaaaleaacqaIYaGmaeqaaaGcbaGaeSO7I0eabaGaemiEaG3aaSbaaSqaaiabdghaXbqabaaaaaGccaGLBbGaayzxaaGaeyypa0ZaamWaaeaafaqabeabbaaaaeaacqWG4baEdaWgaaWcbaGaemyCaehabeaaaOqaaiabdIha4naaBaaaleaacqaIXaqmaeqaaaGcbaGaeSO7I0eabaGaemiEaG3aaSbaaSqaaiabdghaXjabgkHiTiabigdaXaqabaaaaaGccaGLBbGaayzxaaGaeiOla4caaa@61E1@

Note that *σ*_q _is a cyclic function of order q: *σ*_q_^q ^is the identity function on {0,1}^q^.

#### The matrix representation

Any set of pools can be represented by a Boolean matrix, as follows. Each column corresponds to one variable, and each row to one pool. The cell (i,j) is true (value 1) if pool i contains variable j, and false (value 0) otherwise.

#### Example

Consider the n = 9 variables A9
 MathType@MTEF@5@5@+=feaafiart1ev1aaatCvAUfKttLearuWrP9MDH5MBPbIqV92AaeXatLxBI9gBamrtHrhAL1wy0L2yHvtyaeHbnfgDOvwBHrxAJfwnaebbnrfifHhDYfgasaacH8akY=wiFfYdH8Gipec8Eeeu0xXdbba9frFj0=OqFfea0dXdd9vqai=hGuQ8kuc9pgc9s8qqaq=dirpe0xb9q8qiLsFr0=vr0=vr0dc8meaabaqaciaacaGaaeqabaWaaeGaeaaakeaaimaacqWFaeFqdaWgaaWcbaGaeGyoaKdabeaaaaa@394D@ = {A_0_, A_1_,...,A_8_}. The following matrix defines a set of 3 pools:

M0=[100100100010010010001001001]
 MathType@MTEF@5@5@+=feaafiart1ev1aaatCvAUfKttLearuWrP9MDH5MBPbIqV92AaeXatLxBI9gBaebbnrfifHhDYfgasaacH8akY=wiFfYdH8Gipec8Eeeu0xXdbba9frFj0=OqFfea0dXdd9vqai=hGuQ8kuc9pgc9s8qqaq=dirpe0xb9q8qiLsFr0=vr0=vr0dc8meaabaqaciaacaGaaeqabaqabeGadaaakeaacqWGnbqtdaWgaaWcbaGaeGimaadabeaakiabg2da9maadmaabaqbaeqabmqcaaaaaeaacqaIXaqmaeaacqaIWaamaeaacqaIWaamaeaacqaIXaqmaeaacqaIWaamaeaacqaIWaamaeaacqaIXaqmaeaacqaIWaamaeaacqaIWaamaeaacqaIWaamaeaacqaIXaqmaeaacqaIWaamaeaacqaIWaamaeaacqaIXaqmaeaacqaIWaamaeaacqaIWaamaeaacqaIXaqmaeaacqaIWaamaeaacqaIWaamaeaacqaIWaamaeaacqaIXaqmaeaacqaIWaamaeaacqaIWaamaeaacqaIXaqmaeaacqaIWaamaeaacqaIWaamaeaacqaIXaqmaaaacaGLBbGaayzxaaaaaa@4B46@

The pools are {A_0_,A_3_,A_6_} (defined by the first row), {A_1_,A_4_,A_7_} (second row), and {A_2_,A_5_,A_8_} (third row). In fact, this set of pools clearly constitutes a layer.

### Definition of STD

A pooling design is a method to construct a set of pools. When the set of pools can be partitioned into layers (i.e. subsets which each form a partition of the set of variables), the pooling design is said to be "transversal". STD is a transversal pooling design that rearranges the variables from one layer to the next, with two intuitive goals in mind. First, the number of pools in which any pair of variables can occur (i.e. the co-occurrence of variables) should be limited: this is essential for determining the variables' values. The second aim is that the intersections between pools should be of roughly constant size, in order to maximize the mutual information obtained by observing the pools' values and thus increase STD's efficiency.

Given a prime number q with q < n, and k such that k ≤ q+1, STD constructs a set STD(n; q; k) of pools composed of k layers. When k ≤ q, the layers have a uniform construction: they each contain q pools of n/q or (n/q)+1 variables, and are globally interchangeable. In the special case where k = q+1, the q homogeneous layers are supplemented with a singular layer, which has a specific construction and is less regular, yet complements the others nicely. A formal definition of STD(n; q; k) follows.

For every j ∈ {0,...,q}, let *M*_*j *_be a q × n Boolean matrix, defined by its columns *C*_*j*,0_,...,*C*_*j*,*n*-1 _as follows:

C0,0=[10⋮0]
 MathType@MTEF@5@5@+=feaafiart1ev1aaatCvAUfKttLearuWrP9MDH5MBPbIqV92AaeXatLxBI9gBaebbnrfifHhDYfgasaacH8akY=wiFfYdH8Gipec8Eeeu0xXdbba9frFj0=OqFfea0dXdd9vqai=hGuQ8kuc9pgc9s8qqaq=dirpe0xb9q8qiLsFr0=vr0=vr0dc8meaabaqaciaacaGaaeqabaqabeGadaaakeaacqWGdbWqdaWgaaWcbaGaeGimaaJaeiilaWIaeGimaadabeaakiabg2da9maadmaabaqbaeqabqqaaaaabaGaeGymaedabaGaeGimaadabaGaeSO7I0eabaGaeGimaadaaaGaay5waiaaw2faaaaa@3870@, and ∀ i ∈ {0,...,n-1} Cj,i=σqs(i,j)(C0,0)
 MathType@MTEF@5@5@+=feaafiart1ev1aaatCvAUfKttLearuWrP9MDH5MBPbIqV92AaeXatLxBI9gBaebbnrfifHhDYfgasaacH8akY=wiFfYdH8Gipec8Eeeu0xXdbba9frFj0=OqFfea0dXdd9vqai=hGuQ8kuc9pgc9s8qqaq=dirpe0xb9q8qiLsFr0=vr0=vr0dc8meaabaqaciaacaGaaeqabaqabeGadaaakeaacqWGdbWqdaWgaaWcbaGaemOAaOMaeiilaWIaemyAaKgabeaakiabg2da9iabeo8aZnaaDaaaleaacqWGXbqCaeaacqWGZbWCcqGGOaakcqWGPbqAcqGGSaalcqWGQbGAcqGGPaqkaaGccqGGOaakcqWGdbWqdaWgaaWcbaGaeGimaaJaeiilaWIaeGimaadabeaakiabcMcaPaaa@4260@ where:

s(i,j)=∑c=0Γjc⋅⌊iqc⌋
 MathType@MTEF@5@5@+=feaafiart1ev1aaatCvAUfKttLearuWrP9MDH5MBPbIqV92AaeXatLxBI9gBaebbnrfifHhDYfgasaacH8akY=wiFfYdH8Gipec8Eeeu0xXdbba9frFj0=OqFfea0dXdd9vqai=hGuQ8kuc9pgc9s8qqaq=dirpe0xb9q8qiLsFr0=vr0=vr0dc8meaabaqaciaacaGaaeqabaqabeGadaaakeaacqWGZbWCcqGGOaakcqWGPbqAcqGGSaalcqWGQbGAcqGGPaqkcqGH9aqpdaaeWbqaaiabdQgaQnaaCaaaleqabaGaem4yamgaaaqaaiabdogaJjabg2da9iabicdaWaqaaiabfo5ahbqdcqGHris5aOGaeyyXIC9aayWaaeaadaWcaaqaaiabdMgaPbqaaiabdghaXnaaCaaaleqabaGaem4yamgaaaaaaOGaayj84laawUp+aaaa@49EE@ if j < q, and s(i,q)=⌊iqΓ⌋
 MathType@MTEF@5@5@+=feaafiart1ev1aaatCvAUfKttLearuWrP9MDH5MBPbIqV92AaeXatLxBI9gBaebbnrfifHhDYfgasaacH8akY=wiFfYdH8Gipec8Eeeu0xXdbba9frFj0=OqFfea0dXdd9vqai=hGuQ8kuc9pgc9s8qqaq=dirpe0xb9q8qiLsFr0=vr0=vr0dc8meaabaqaciaacaGaaeqabaqabeGadaaakeaacqWGZbWCcqGGOaakcqWGPbqAcqGGSaalcqWGXbqCcqGGPaqkcqGH9aqpdaGbdaqaamaalaaabaGaemyAaKgabaGaemyCae3aaWbaaSqabeaacqqHtoWraaaaaaGccaGLWJVaay5+4daaaa@3E06@, where the semi-bracket denotes the integer part.

Let *L*(*j*) be the set of pools of which *M*_*j *_is the matrix representation. Note that each column *C*_*j,i *_has exactly one occurrence of '1' and (q-1) occurrences of '0'. The index of the '1' identifies the (single) pool of *L*(*j*) which contains variable A_i_. Therefore, in a given set of pools *L*(*j*), each variable is present in exactly one pool, that is to say *L*(*j*) constitutes a partition of An
 MathType@MTEF@5@5@+=feaafiart1ev1aaatCvAUfKttLearuWrP9MDH5MBPbIqV92AaeXatLxBI9gBamrtHrhAL1wy0L2yHvtyaeHbnfgDOvwBHrxAJfwnaebbnrfifHhDYfgasaacH8akY=wiFfYdH8Gipec8Eeeu0xXdbba9frFj0=OqFfea0dXdd9vqai=hGuQ8kuc9pgc9s8qqaq=dirpe0xb9q8qiLsFr0=vr0=vr0dc8meaabaqaciaacaGaaeqabaWaaeGaeaaakeaaimaacqWFaeFqdaWgaaWcbaWexLMBbXgBcf2CPn2qVrwzqf2zLnharCGvLjhzH5wyaGqbaiaa+5gaaeqaaaaa@43C8@: *L*(*j*) is a layer.

Finally, for k ∈ {1,2,...,q+1}, STD(n; q; k) is defined as: STD(n; q; k)=∪j=0k−1L(j)
 MathType@MTEF@5@5@+=feaafiart1ev1aaatCvAUfKttLearuWrP9MDH5MBPbIqV92AaeXatLxBI9gBaebbnrfifHhDYfgasaacH8akY=wiFfYdH8Gipec8Eeeu0xXdbba9frFj0=OqFfea0dXdd9vqai=hGuQ8kuc9pgc9s8qqaq=dirpe0xb9q8qiLsFr0=vr0=vr0dc8meaabaqaciaacaGaaeqabaqabeGadaaakeaacqqGtbWucqqGubavcqqGebarcqGGOaakcqqGUbGBcqqG7aWocqqGGaaicqqGXbqCcqqG7aWocqqGGaaicqqGRbWAcqGGPaqkcqGH9aqpdaWeWbqaaiabdYeamjabcIcaOiabdQgaQjabcMcaPaWcbaGaemOAaOMaeyypa0JaeGimaadabaGaem4AaSMaeyOeI0IaeGymaedaniablQIivbaaaa@4710@.

#### Example

Consider again the variables A9
 MathType@MTEF@5@5@+=feaafiart1ev1aaatCvAUfKttLearuWrP9MDH5MBPbIqV92AaeXatLxBI9gBamrtHrhAL1wy0L2yHvtyaeHbnfgDOvwBHrxAJfwnaebbnrfifHhDYfgasaacH8akY=wiFfYdH8Gipec8Eeeu0xXdbba9frFj0=OqFfea0dXdd9vqai=hGuQ8kuc9pgc9s8qqaq=dirpe0xb9q8qiLsFr0=vr0=vr0dc8meaabaqaciaacaGaaeqabaWaaeGaeaaakeaaimaacqWFaeFqdaWgaaWcbaWexLMBbXgBcf2CPn2qVrwzqf2zLnharCGvLjhzH5wyaGqbaiaa+5gaaeqaaaaa@43C8@, and let q = 3 (hence Γ = 1). *M*_0 _is as defined above, and *M*_1_, *M*_2_, *M*_3 _are:

M1=[100001010010100001001010100],M2=[100010001010001100001100010],M3=[111000000000111000000000111].
 MathType@MTEF@5@5@+=feaafiart1ev1aaatCvAUfKttLearuWrP9MDH5MBPbIqV92AaeXatLxBI9gBaebbnrfifHhDYfgasaacH8akY=wiFfYdH8Gipec8Eeeu0xXdbba9frFj0=OqFfea0dXdd9vqai=hGuQ8kuc9pgc9s8qqaq=dirpe0xb9q8qiLsFr0=vr0=vr0dc8meaabaqaciaacaGaaeqabaqabeGadaaakqaabeqaaiabd2eannaaBaaaleaacqaIXaqmaeqaaOGaeyypa0ZaamWaaeaafaqabeWajaaaaaqaaiabigdaXaqaaiabicdaWaqaaiabicdaWaqaaiabicdaWaqaaiabicdaWaqaaiabigdaXaqaaiabicdaWaqaaiabigdaXaqaaiabicdaWaqaaiabicdaWaqaaiabigdaXaqaaiabicdaWaqaaiabigdaXaqaaiabicdaWaqaaiabicdaWaqaaiabicdaWaqaaiabicdaWaqaaiabigdaXaqaaiabicdaWaqaaiabicdaWaqaaiabigdaXaqaaiabicdaWaqaaiabigdaXaqaaiabicdaWaqaaiabigdaXaqaaiabicdaWaqaaiabicdaWaaaaiaawUfacaGLDbaacqGGSaalaeaacqWGnbqtdaWgaaWcbaGaeGOmaidabeaakiabg2da9maadmaabaqbaeqabmqcaaaaaeaacqaIXaqmaeaacqaIWaamaeaacqaIWaamaeaacqaIWaamaeaacqaIXaqmaeaacqaIWaamaeaacqaIWaamaeaacqaIWaamaeaacqaIXaqmaeaacqaIWaamaeaacqaIXaqmaeaacqaIWaamaeaacqaIWaamaeaacqaIWaamaeaacqaIXaqmaeaacqaIXaqmaeaacqaIWaamaeaacqaIWaamaeaacqaIWaamaeaacqaIWaamaeaacqaIXaqmaeaacqaIXaqmaeaacqaIWaamaeaacqaIWaamaeaacqaIWaamaeaacqaIXaqmaeaacqaIWaamaaaacaGLBbGaayzxaaGaeiilaWcabaGaemyta00aaSbaaSqaaiabiodaZaqabaGccqGH9aqpdaWadaqaauaabeqadKaaaaaabaGaeGymaedabaGaeGymaedabaGaeGymaedabaGaeGimaadabaGaeGimaadabaGaeGimaadabaGaeGimaadabaGaeGimaadabaGaeGimaadabaGaeGimaadabaGaeGimaadabaGaeGimaadabaGaeGymaedabaGaeGymaedabaGaeGymaedabaGaeGimaadabaGaeGimaadabaGaeGimaadabaGaeGimaadabaGaeGimaadabaGaeGimaadabaGaeGimaadabaGaeGimaadabaGaeGimaadabaGaeGymaedabaGaeGymaedabaGaeGymaedaaaGaay5waiaaw2faaiabc6caUaaaaa@8B32@

The corresponding layers of pools are the following:

Layer 0: *L*(0) = {{A_0_,A_3_,A_6_}, {A_1_,A_4_,A_7_}, {A_2_,A_5_,A_8_}}

Layer 1: *L*(1) = {{A_0_,A_5_,A_7_}, {A_1_,A_3_,A_8_}, {A_2_,A_4_,A_6_}}

Layer2: *L*(2) = {{A_0_,A_4_,A_8_}, {A_1_,A_5_,A_6_}, {A_2_,A_3_,A_7_}}

Layer3: *L*(3) = {{A_0_,A_1_,A_2_}, {A_3_,A_4_,A_5_}, {A_6_,A_7_,A_8_}}.

STD(9; 3; 2) is the following set of pools: STD(9; 3; 2) = *L*(0) ∪ *L*(1).

#### Remark

The method builds at most q+1 layers: indeed, if we discard the last particular layer *L*(*q*) and attempt to extend the STD construction to any j, it becomes cyclic of order q: for every j, *L*(*j*+*q*) = *L*(*j*).

## Results (2): properties of STD

In this section, we establish an important theorem, leading to three corollaries which show that STD constitutes a solution to the pooling problem described in the introduction, and that it can be used to detect and correct noisy observations. We then establish another property of STD, which is noteworthy albeit not directly related to the pooling problem.

### Co-occurrence of variables

So far we have considered the variables that are contained in a given pool. Dually, we may consider the set of pools that contain a given variable. For k ∈ {1,2,...,q+1}, we will note *pools*_*k*_(*i*) the set of pools of STD(n; q; k) that contain variable A_i_:

∀ i ∈ {0,...,n-1}, *pools*_*k*_(*i*) = {p ∈ STD(n; q; k) | A_i _∈ p}.

#### Theorem 1

Recall that q is prime.

∀ i_1_, i_2 _∈ {0,...,n-1}, [i_1_≠i_2_] ⇒ [Card(*pools*_*q*+1_(*i*_1_) ∩ *pools*_*q*+1_(*i*_2_)) ≤ Γ(q,n)].

#### Proof

see Methods section.

#### Example

Consider again the example n = 9, q = 3, k = 4, for which the layers *L*(0), *L*(1), *L*(2), and *L*(3) are known (see above). The set of pools containing A_0 _is: *pools*_4_(0) = {{A_0_,A_3_,A_6_},{A_0_,A_5_,A_7_},{A_0_,A_4_,A_8_},{A_0_,A_1_,A_2_}}.

One can easily see that A_0 _is present exactly once with each other variable. In fact, each pair of variables is present in exactly 1 (= Γ(3,9)) pool, in conformity with theorem 1.

#### Remark

The property holds a fortiori when k < q+1, i.e. when considering STD(n; q; k) instead of STD(n; q; q+1).

### A solution to the pooling problem

#### Corollary 1

Let t be an integer such that t·Γ(q,n) ≤ q. Let k = t·Γ+1, and consider the set of pools STD(n; q; k). Suppose that the value of each pool has been observed, and that there are at most t positive variables in An
 MathType@MTEF@5@5@+=feaafiart1ev1aaatCvAUfKttLearuWrP9MDH5MBPbIqV92AaeXatLxBI9gBamrtHrhAL1wy0L2yHvtyaeHbnfgDOvwBHrxAJfwnaebbnrfifHhDYfgasaacH8akY=wiFfYdH8Gipec8Eeeu0xXdbba9frFj0=OqFfea0dXdd9vqai=hGuQ8kuc9pgc9s8qqaq=dirpe0xb9q8qiLsFr0=vr0=vr0dc8meaabaqaciaacaGaaeqabaWaaeGaeaaakeaaimaacqWFaeFqdaWgaaWcbaWexLMBbXgBcf2CPn2qVrwzqf2zLnharCGvLjhzH5wyaGqbaiaa+5gaaeqaaaaa@43C8@. Then, in the absence of noise (i.e., if all pool values are correctly observed), the value of every variable can be identified.

#### Proof

Consider the following algorithm, which tags variables as negative or positive.

Algorithm 1: all the variables present in at least one negative pool are tagged negative; any variable present in at least one positive pool where all other variables have been tagged negative, is tagged positive.

We show that this algorithm correctly identifies the value of each and every variable.

Let A_i _be a negative variable. A_i _is present in exactly k pools: one pool in each layer. Theorem 1 asserts that no variable other than A_i _is present in more than Γ of these t·Γ+1 pools. Therefore, since at most t variables are positive, A_i _is present in at least one pool where no positive variable is present. Consequently, examination of this pool yields a negative answer (since all observations are correct), which leads algorithm 1 to tag A_i _negative. This shows that every negative variable is correctly tagged as such.

Now let A_i _be a positive variable. Since we suppose here that there are no observational errors, all pools containing A_i _are positive: A_i _is not tagged negative. Again according to theorem 1, no other variable is present in more than Γ of these t·Γ+1 pools. Therefore, since there are at most t-1 other positive variables, A_i _is present in at least Γ+1 positive pools where all other variables are negative, and are tagged negative according to the above. This shows that every positive variable is tagged correctly and uniquely.

Finally, since every positive pool must contain at least one positive variable, and since no positive variable is tagged negative, we can conclude that no negative variable can be tagged positive (in addition to its negative tag): every negative variable is also uniquely tagged. This completes the corollary's proof.

#### Example

Consider again our example STD(9; 3; 2) = {{A_0_,A_3_,A_6_}, {A_1_,A_4_,A_7_}, {A_2_,A_5_,A_8_}, {A_0_,A_5_,A_7_}, {A_1_,A_3_,A_8_}, {A_2_,A_4_,A_6_}}.

Let t = 1, and suppose that a single variable in A9
 MathType@MTEF@5@5@+=feaafiart1ev1aaatCvAUfKttLearuWrP9MDH5MBPbIqV92AaeXatLxBI9gBamrtHrhAL1wy0L2yHvtyaeHbnfgDOvwBHrxAJfwnaebbnrfifHhDYfgasaacH8akY=wiFfYdH8Gipec8Eeeu0xXdbba9frFj0=OqFfea0dXdd9vqai=hGuQ8kuc9pgc9s8qqaq=dirpe0xb9q8qiLsFr0=vr0=vr0dc8meaabaqaciaacaGaaeqabaWaaeGaeaaakeaaimaacqWFaeFqdaWgaaWcbaGaeGyoaKdabeaaaaa@394D@ is positive. For reasons of symmetry, the name of that variable is inconsequent: all are equivalent. Let us suppose that the only positive variable is A_8_. Then pools {A_0_,A_3_,A_6_}, {A_1_,A_4_,A_7_}, {A_0_,A_5_,A_7_}, and {A_2_,A_4_,A_6_} are negative, which shows that variables A_0_, A_1_,...,A_7 _are negative; and pools {A_2_,A_5_,A_8_} and {A_1_,A_3_,A_8_} are positive, which each prove that A_8 _is positive (given that A_2_, A_5_, A_1 _and A_3 _have been shown to be negative).

#### Remark

If more than t variables are positive, this fact is revealed: clearly, at most n - (t+1) variables are tagged negative, contrary to when there are at most t positives. In fact, all tags produced by the above algorithm are still correct, but some variables may not be tagged at all: these variables are called "unresolved", or "ambiguous". It would be interesting to know how many ambiguous variables are to be expected, but this is a very hard problem to study analytically, particularly when one takes into account experimental noise. Instead, this issue can be suitably approached by computer simulation.

### Dealing with noise: error correction

As stated in the introduction, pooling designs have an intrinsic potential for noise-correction, due to the redundancy of variables. In the case of STD, this potential can be taken advantage of by simply testing a few extra layers of pools and using a modified algorithm, as shown here.

#### Corollary 2

Let t and E be integers such that t·Γ(q,n)+2·E ≤ q, and let k = t·Γ+2·E+1. Consider the set of pools STD(n; q; k), and suppose that the value of each pool has been observed. Furthermore, suppose that there are at most t positive variables in An
 MathType@MTEF@5@5@+=feaafiart1ev1aaatCvAUfKttLearuWrP9MDH5MBPbIqV92AaeXatLxBI9gBamrtHrhAL1wy0L2yHvtyaeHbnfgDOvwBHrxAJfwnaebbnrfifHhDYfgasaacH8akY=wiFfYdH8Gipec8Eeeu0xXdbba9frFj0=OqFfea0dXdd9vqai=hGuQ8kuc9pgc9s8qqaq=dirpe0xb9q8qiLsFr0=vr0=vr0dc8meaabaqaciaacaGaaeqabaWaaeGaeaaakeaaimaacqWFaeFqdaWgaaWcbaWexLMBbXgBcf2CPn2qVrwzqf2zLnharCGvLjhzH5wyaGqbaiaa+5gaaeqaaaaa@43C8@, and that there are at most E observation errors. Then, all errors can be detected and corrected, and the value of every variable can be identified.

#### Proof

Consider the following tagging algorithm.

Algorithm 2: all the variables present in at least E+1 negative pools are tagged negative; any variable present in at least E+1 positive pools where all other variables have been tagged negative, is tagged positive.

The proof is similar to that of corollary 1: we show that algorithm 2 correctly and uniquely tags every variable. In this case, theorem 1 shows that each negative variable is necessarily present in at least 2·E+1 negative pools. Since there are at most E observation errors, it follows that at least E+1 of these negative pools are correctly observed. Therefore, algorithm 2 tags all negative variables as such. In addition, at most E pools containing a positive variable can be observed negative; hence no positive variable is tagged negative. Finally, since at most E pools containing only negative variables can be observed positive, no negative variable is tagged positive:every negative variable is correctly and uniquely tagged.

Conversely, a positive variable A_i _appears in at least t·Γ+E+1 positive pools (since there are at most E errors), of which at most (t-1)·Γ contain at least one other positive variable (according to theorem 1). Therefore A_i _is present in at least (t·Γ+E+1) - (t-1)·Γ = Γ+E+1 positive pools where all other variables are negative. Since these negative variables have been correctly tagged as such (as shown above), A_i _is tagged positive. This shows that algorithm 2 also correctly and uniquely tags all positive variables.

Finally, any observation which is contradictory with the obtained tagging is necessarily erroneous. In other words, false negative and false positive observations are identified.

#### Remark

Few restrictions are imposed when choosing the value of the parameter q: it must simply be a prime number smaller than n. Consequently, STD can be used successfully even when very high noise levels are expected, by picking a large value for q. Of course, as is to be expected in low signal-to-noise situations, this high corrective power comes at the price of lower compression performance, since larger q values mean more pools per layer.

Corollary 2 does not distinguish between the two types of errors: false positives and false negatives. If we consider them separately, the corrective power of STD can actually be improved twofold, as shown below.

#### Corollary 3

Let t and E be integers such that t·Γ(q,n)+2·E ≤ q, and let k = t·Γ+2·E+1. Consider the set of pools STD(n; q; k), and suppose that the value of each pool has been observed. Furthermore, suppose that there are at most t positive variables in An
 MathType@MTEF@5@5@+=feaafiart1ev1aaatCvAUfKttLearuWrP9MDH5MBPbIqV92AaeXatLxBI9gBamrtHrhAL1wy0L2yHvtyaeHbnfgDOvwBHrxAJfwnaebbnrfifHhDYfgasaacH8akY=wiFfYdH8Gipec8Eeeu0xXdbba9frFj0=OqFfea0dXdd9vqai=hGuQ8kuc9pgc9s8qqaq=dirpe0xb9q8qiLsFr0=vr0=vr0dc8meaabaqaciaacaGaaeqabaWaaeGaeaaakeaaimaacqWFaeFqdaWgaaWcbaWexLMBbXgBcf2CPn2qVrwzqf2zLnharCGvLjhzH5wyaGqbaiaa+5gaaeqaaaaa@43C8@, and that there are at most E false positive and E false negative observations. Then, all errors can be detected and corrected, and the value of every variable can be identified.

#### Proof

The proof of corollary 2 can be directly replicated, and shows that algorithm 2 still tags all variables uniquely and correctly. Indeed, since there are at most E false positives, every negative variable is tagged as such; and since there are at most E false negatives, no positive variable is tagged negative. In addition, no negative variable is tagged positive: this results from the facts that there are at most E false positives and that no positive variable is tagged negative. Finally, given that every negative variable is tagged negative, we can conclude that every positive variable is tagged positive as long as there are less than E+Γ+1 false negatives.

### Error detection

If algorithm 2 tags some variables twice or not at all, or if it tags more than t variables as positive, or if it identifies more than E false positives or false negatives, then we know that the conditions for corollaries 2 and 3 are not satisfied. In this case the obtained tags may be incorrect, but one is aware of the situation. However, if enough excess errors are present, the tags can be wrong while seeming to satisfy one of the corollaries' hypotheses; in this case, the mistake is not detected. This leads to the following important question: in general, assuming there are at most t positives, how many errors can be detected?

Examining the proof of corollary 3, if there are at most E false positives and up to E+Γ false negatives, every variable is correctly tagged, although some variables may be tagged twice (i.e. both positive and negative). It follows that to avoid detection, there must be at least E+Γ+1 false negatives, or at least E+1 false positives. In fact, E+Γ+1 false negatives can successfully remain undetected. On the other hand, if there are E+1 false positives, a negative variable may seem positive with only E fictitious false negatives; but this would lead to t+1 putative positive variables, hence detection is in fact not avoided. A detailed analysis shows that escaping detection in this case actually requires either Γ extra false positives, or 2·E+1 additional errors among which at least E+1 are false negatives. Overall, ignoring the errors' types, we conclude that the detection of min(3·E+1, E+Γ) errors is guaranteed. Typically Γ is 2 or 3, hence this guarantee is not very strong; but it corresponds to a rare worst case scenario, and in practice many more errors can usually be detected.

**Figure 1:**
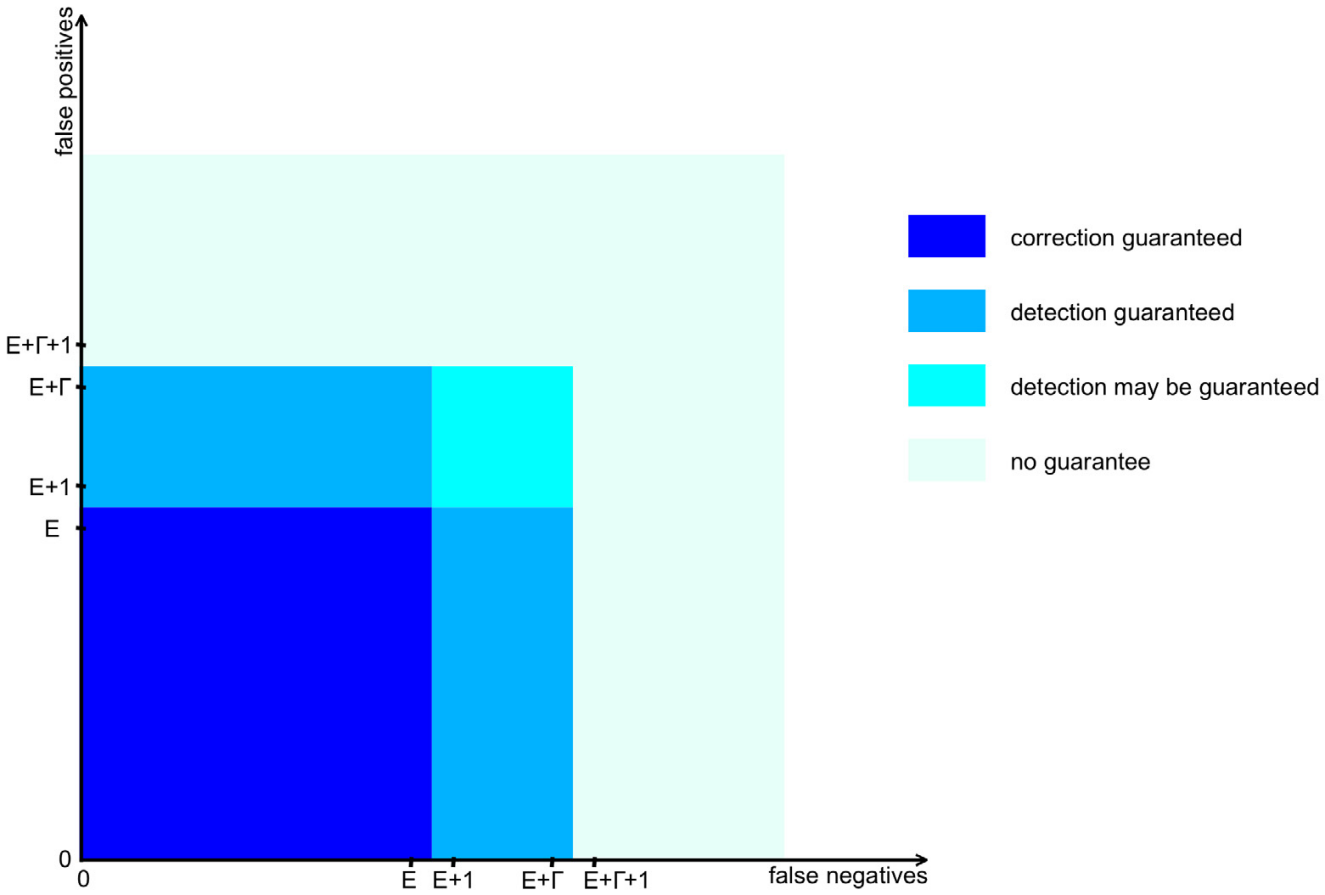
**Guaranteed error correction and detection properties of STD**. An experimenter, expecting up to t positives and E errors, chooses a satisfactory prime number q and builds the set of pools STD(n; q; t·Γ+2·E+1), as specified in corollary 2. Recall that n is the total number of variables and Γ is the compression power, i.e. the smallest *γ *such that q^γ+1 ^≥ n. This figure summarizes the behavior of these pools when the actual number of errors exceeds E, and distinguishes between the two types of errors: false positives and false negatives. In the dark blue region, all errors are detected and corrected. In the intermediate blue rectangles, correction is not guaranteed but detection is: in an unfavorable conformation of positives and errors, correction of all errors may fail, but this failure cannot go unnoticed, and the user can therefore plan additional experiments. In the cyan square, detection is usually also guaranteed, except if E is very small (E < 2·Γ-1): in this case, the line y = 3·E+1-x splits the square in two, and detection is only guaranteed in the bottom left portion, where the total number of errors is at most 3·E+1. Finally, in the outer pale cyan zone, no guarantee is provided.

The error correction and detection properties of STD are summarized in Figure 1. From another angle, it is interesting to know what happens if more than t variables are positive. As long as there are at most E errors, all tags produced by algorithm 2 are still correct, although some variables may not be tagged (i.e., they are unresolved). Therefore the occurrence of more than t positives is detected, as in the noiseless case. However, if there are both more than E errors and more than t positives, problems may occur and escape detection (e.g., a positive variable might be "mis-tagged" as negative). Some of these problems reflect the natural limits of the STD pools, and can only be avoided by using different STD parameters; but some result from the rigidity of algorithm 2. In real applications where the number of positives and errors will probably exceed t and E in at least a few instances, more sophisticated algorithms should be used.

### Even redistribution of variables

We have just shown that STD constitutes a solution to the pooling problem in the presence of experimental noise. Although it digresses from the main focus of this paper, the following theorem provides an interesting characterization of STD, basically showing that the STD layers work well together, information-wise.

#### Theorem 2

Let m ≤ k ≤ q and consider a set of m pools {P_1_,...,P_m_} ⊂ STD(n; q; k), each belonging to a different layer. Then:

λm≤|∩h=1mPh|≤λm+1,whereλm=∑c=mΓ[⌊n−1qc⌋%q]⋅qc−m.
 MathType@MTEF@5@5@+=feaafiart1ev1aaatCvAUfKttLearuWrP9MDH5MBPbIqV92AaeXatLxBI9gBaebbnrfifHhDYfgasaacH8akY=wiFfYdH8Gipec8Eeeu0xXdbba9frFj0=OqFfea0dXdd9vqai=hGuQ8kuc9pgc9s8qqaq=dirpe0xb9q8qiLsFr0=vr0=vr0dc8meaabaqaciaacaGaaeqabaqabeGadaaakeaafaqabeqadaaabaacciGae83UdW2aaSbaaSqaaGqaciab+1gaTbqabaGccqGHKjYOdaabdaqaamaauahabaGaemiuaa1aaSbaaSqaaiabdIgaObqabaaabaGaemiAaGMaeyypa0JaeGymaedabaGaemyBa0ganiablMIijbaakiaawEa7caGLiWoacqGHKjYOcqWF7oaBdaWgaaWcbaGae4xBa0gabeaakiabgUcaRiabigdaXiabcYcaSaqaaiabbEha3jabbIgaOjabbwgaLjabbkhaYjabbwgaLbqaaiab=T7aSnaaBaaaleaacqGFTbqBaeqaaOGaeyypa0ZaaabCaeaadaWadaqaamaagmaabaWaaSaaaeaacqWGUbGBcqGHsislcqaIXaqmaeaacqWGXbqCdaahaaWcbeqaaiabdogaJbaaaaaakiaawcp+caGL7JpacqGGLaqjcqWGXbqCaiaawUfacaGLDbaacqGHflY1cqWGXbqCdaahaaWcbeqaaiabdogaJjabgkHiTiabd2gaTbaaaeaacqWGJbWycqGH9aqpcqWGTbqBaeaacqqHtoWra0GaeyyeIuoaaaGccqGGUaGlaaa@702E@

#### Proof

see Methods section.

#### Remarks

1. *λ*_*m *_depends only on m and not on the choice of P_1_,...,P_m_; hence this theorem can be expressed simply as follows: each pool is redistributed evenly in every other layer, and furthermore the intersection between any two or more pools from different layers is also redistributed evenly in the remaining layers. This property is very interesting because it means that knowing that any given pool is positive doesn't bring any information regarding which pools of another layer will be positive; hence, the information content of the other layers remains high.

2. Note that the theorem specifies k ≤ q rather than q+1: the last layer that can be built with STD, *L*(*q*), is particular and does not satisfy theorem 2.

## Discussion

To evaluate and compare pooling designs, a fair performance measure is needed. A widely-used and reasonable choice consists in considering the number of pools required to guarantee the correction of all errors and the identification of all variables' values: we call this the "guarantee requirement". This criterion is used here to study the behavior and performance of STD, and to compare it to the main published deterministic error-correcting pooling designs. Since most authors do not distinguish between false positives and false negatives, we only consider here the error correction power of STD as stated in corollary 2, rather than the stronger result expressed in corollary 3.

### Guaranteed performance of STD

We define the "gain" of a design as the ratio between the number of variables and the number of pools: n/v. The gain is called "guaranteed gain" if the guarantee requirement is satisfied. This measure is particularly useful for comparing settings where n varies.

Given the specifications of an application, i.e. values for n (total number of objects to be tested), t (number of expected positives), and E (expected number of errors to be corrected), STD can propose many sets of pools, by selecting various values for the parameter q and setting the number of layers k accordingly (as specified by corollary 2). These pool sets are of different sizes, but all satisfy the guarantee requirement. The optimal choice, q_opt_, is the one with maximum guaranteed gain. Let q_min _be the smallest possible q such that t·Γ(q,n)+2·E ≤ q, and let Γ_max _= Γ(q_min_,n). At a fixed value for Γ, the number of layers k necessary to satisfy the guarantee requirement is constant; therefore the best gain at fixed Γ is always obtained with the smallest q whose compression is Γ. It follows that q_opt _can be identified easily by finding the smallest q for each value of Γ in {1,...,Γ_max_}, and calculating the corresponding gain. In practice we often have q_opt _= q_min_, but this is not compulsory, as illustrated by Table 1 in the case n = 10000, t = 5, E = 0.

The above method allows to easily calculate the best guaranteed gain that STD can offer, in any specified (n,t,E) setting. Therefore, the behavior of STD can be studied under various angles. In particular, one interesting approach consists in using fixed values for t and E, and studying the evolution of the best guaranteed gain (obtained using q_opt_) when n increases. For example, Table 2 displays the number of pools necessary to identify three positives and correct two errors, when the number of variables ranges from 100 to 10^6^. When n increases, the gain increases substantially and fairly regularly: it is multiplied by a factor ranging from 6 to 9 every time n gains an order of magnitude. Note that in a real application, the fact that the pool sizes are generally constrained by practical considerations can result in forcing to use values of q > q_opt _and hence limit the gain.

### Comparison with previous work

In this section, after a brief overview of the known construction methods, we compare STD, in terms of flexibility and performance under the guarantee requirement, to the main published error-correcting deterministic pooling designs. In general, the guaranteed gains can be difficult to compare analytically, because the numbers of pools and variables can be defined by formulas that are often rather involved. However, each paper describing a new design typically holds a numerical example, which would hardly be disadvantageous to the described design. Therefore, when the methods cannot be easily compared, it seems fair to use each paper's numerical example for comparison with STD. Note that the guarantee requirement cannot be satisfied by random designs [e.g. [8]], which are consequently not studied here.

Detailed reviews of deterministic pooling designs can be found in [7,9,10], and we will only very briefly recapitulate them here. Broadly speaking, there are three main construction methods: set packings, transversal designs, and direct constructions. In fact, the non-adaptive pooling problem is strongly connected to the problem of constructing superimposed codes [11], which was analyzed forty years ago to deal with the questions of representing rare document attributes in an information retrieval system and of assigning channels to relieve congestion in shared communications bands. The focus is different: each variable is seen as a code word and the goal is to maximize the number of code words n at fixed length v rather than the other way around; and these problems were noiseless, contrary to our own situation where error-correction is critical. Yet [11] had already suggested constructions of superimposed codes based on set packings, as well as constructions based on q-nary codes (which are in fact transversal designs) and on compositions of q-nary codes (which are not transversal anymore, and are more compact). Set packings, such as the designs presented in [12], can yield very efficient designs, but are mainly limited to t ≤ 2 [7]. Transversal designs include the well-known grid (or row-and-column) design. This design is initially limited to identifying a single positive in the absence of noise, and is not very efficient, but it has been improved in two directions: hypercube designs [13] generalize it by considering higher dimension grids, and various methods [e.g. [14]] have been proposed to build several "synergical" grids that work well together. Finally, some authors have proposed direct constructions of error-correcting pooling designs [15,16].

Note that STD, although directly constructed, is in fact a transversal design. Furthermore, STD can be seen as a constructive definition of a q-nary code as proposed by [11], i.e. a concatenated code where the inner code is simply the unary code, and the outer code has some similarities with a Reed-Solomon code [17]. Yet although related, the methods are clearly different: for example, STD doesn't produce useful pools if q is a prime power; on the other hand, STD allows to build up to q+1 layers, whereas the Reed-Solomon based construction can only build up to q-1. Furthermore, STD produces efficient pools independently of the number of variables n, contrary to the Reed-Solomon approach where one is faced with the difficult problem of choosing a good subset of code words except for some n values. The relationship between the two approaches requires further investigations.

#### Set packing designs

Regarding set packing designs, the main results taking into account error-correction are presented in [12]. The authors exhibit Steiner designs that can identify up to t = 2 positives and in some instances correct many errors, and prove that these designs are optimal when the construction is possible (it is only possible for very specific (n,E) values). When these optimal designs exist, they are more efficient than STD. The same authors describe a real-world application in [18], where the goal is to screen a clone map with n = 1530 and t = 2. They start off with a design that can deal with 4368 variables while satisfying the guarantee requirement for t = 2 and E = 0. None of the optimal designs from [12] can be used, but this initial design is also based on a Steiner system and remains very efficient. The authors then select 1530 of the 4368 variables to serve as clones in their experiment. This was presumably done because Steiner systems, even outside the optimality conditions of [12], are not known for arbitrary values of n. Although this reduces the resulting designs' performance, they remain efficient and obviously still satisfy the guarantee requirement. Additionally, this strategy reduces the sizes of pools, providing increased robustness (e.g., some information can still be obtained if, exceptionally, three objects are positive), and complying with the application-imposed pool size constraints. In the example, n = 1530 and t = 2, and the authors propose two designs: one with 65 pools of approximately 118 clones each, and one with 54 pools of 142 clones. These numbers are very close to what would be recommended with STD: we could propose STD(1530; 13; 5) which has 65 pools of 118 clones, or STD(1530; 7; 7) with 49 pools of 218 clones. Note that although STD(1530; 13; 5) has the same number of pools and pool size as the first design proposed in [18], they are in fact different: the latter is obtained by random sampling from the Steiner design. All of these designs guarantee the identification of 2 positives in the absence of noise. Furthermore, although noise-tolerance is not guaranteed in any of them, simulations we have performed suggest that substantial error-rates can be corrected in the STD designs, as is the case in the others. Therefore these designs and STD appear to achieve very similar performances on these examples. However, it is important to note that the only Steiner systems proven to be optimal concern specific instances of the t = 1 and t = 2 cases. In more general circumstances, designs derived from Steiner systems are not optimal, and their performance depends on the problem specification (i.e. n, t, E values). For example, considering the n = 10000, t = 5, E = 0 problem discussed above and in Table 1, the smallest Steiner system that we could identify (based on [19]) is S(3,24,530), which comprises 530 pools. In addition, there is no clear method for choosing the Steiner system best suited to a given problem specification: although we have searched extensively, we cannot be certain that no better Steiner system exists for this example. In contrast, finding the optimal STD parameters is straight-forward, as explained in the previous section. In this case STD proposes a solution comprising 253 pools.

#### Transversal designs

An interesting generalization of the grid design is described in [13]. The authors propose to array the variables in a D-dimensional cube, instead of the 2 dimensions used in the standard grid design. Furthermore, they advise that the length of the cube's side be chosen prime: let us denote it q. A pool is then obtained from each hyperplane, so that the D-dimensional cube yields D layers of q pools, each comprising up to n/q variables. To obtain more layers, the authors propose a criterion to construct "efficient transforming matrices" that produce additional cubes, where variables are as shuffled as possible; in fact, the purpose of their "efficiency" criterion is identical to the "co-occurrence of variables" property satisfied by STD (theorem 1). Seen like this, their system is clearly related to STD: D is Γ+1, and although the authors do not investigate their design's behavior under the guarantee requirement, corollaries 1 and 2 can in essence be applied. Furthermore, when the cube is "full", i.e. when n = q^D^, their pools satisfy an analog of theorem 2 (i.e. they are "information-efficient" in some sense). Note that this cannot be the case when q is arbitrary; this may explain why the authors limit their options for q to the smallest primes larger than n^1/D^, for each D value. However, each D-dimensional cube provides only D layers, and the proposed criterion for building additional cubes is not systematic, so that the total number of layers that can be built is unclear but seems much smaller than with STD. In addition, the authors don't take observational noise into account (they do talk of "false positives", but are really referring to what we call ambiguous variables). For these reasons, we cannot rigorously compare the designs under the guarantee requirement, but in general the fact that STD can build more layers is clearly favorable, since it allows dealing with a greater number of positives and/or errors at any chosen q value. In a numerical example concerning the screening of the CEPH YAC library, n = 72000 and the authors argue that the optimal dimension and side length to use are D = 3 and q = 43, respectively. They then exhibit a set of transforming matrices that allows the construction of at most 3 additional cubes, yielding a total of 12 layers. By contrast, using the same values for D and q, STD can build up to 44 layers, which all satisfy the efficiency criterion. We believe that some of these extra layers could prove valuable, especially when allowing for experimental noise. In addition, smaller values for q can be used with STD (while still being information-efficient in the sense of theorem 2), although simulations would be necessary to choose the best value.

Two other transversal pooling designs, which generalize the grid design by providing additional 2-dimensional grids, are described in [14]: the "Union Jack" and the RCF designs. In essence, they are very similar to STD when Γ = 1: writing q = √n, they allow the construction of up to q+1 layers of pools (where each layer contains q pools of size q) which satisfy the property that any pair of variables appears in at most one pool. Theorem 1 shows that this property, known as the "unique colinearity" condition, is in fact verified by STD when Γ = 1 (in accord with q = √n). We can observe that these designs, as well as STD when Γ = 1, are maximal under this condition, since each pair of variables is in fact present in exactly one pool. Corollaries 1 and 2 can be applied, and show that they allow the identification of up to t positives while correcting E observation errors, provided that t+2·E+1 ≤ q+1. The performance of the designs from [14] is therefore identical to that of STD when Γ = 1. However, STD is superior to these designs in two respects. First, their constructions are only possible if q is prime and q≡5 mod 6 (using the RCF construction), or if q is prime and q≡3 mod 4 (with the Union Jack design). By contrast, STD only requires that q is prime. Second, STD can be used with any compression power, rather than being limited to Γ = 1. This flexibility is an advantage, because STD can be customized to suit more applications. Notably, when the fraction of positives is small, the Union Jack and RCF designs perform less well: the pools are too small, and observing that a pool is negative brings little information. By contrast, pools in STD can be very large (when choosing a small q), so that every observation is informative. To illustrate this point, let us consider the numerical example of [16] discussed below, where the fraction of positives is particularly low (n = 18,918,900 and t is 2 or 9). The best usable design from [14] would be a Union Jack with q = 4363, and would require a total of 13,089 pools for 2 positives – 77 times more than STD – and 43,630 pools to guarantee the identification of 9 positives – 32 times more than STD.

#### Direct constructions

In [15], the author proposes a direct construction allowing the detection of an arbitrary number of positives. Although this design is not very efficient under the guarantee requirement, the author shows in [20] that the pools designed for detecting 2 positives allow with high probability the detection of more positives. A numerical example, presented in [9], is the following. If n = 10^6 ^and t = 5, using 946 pools guarantees the identification of 2 positives and successfully identifies up to 5 positives with probability 97.1%. In comparison, under the guarantee requirement (i.e. with probability 100%), STD(n; 11; 11) contains 121 pools and identifies 2 positives, and STD(n; 23; 21), which comprises 483 pools, guarantees the identification of up to 5 positives.

Finally, another group [16] described two new classes of non-adaptive pooling designs, which allow the detection of any number of positives and the correction of half as many errors. Following the idea from [20], they also show that their designs for t = 2 have high probabilities of being successful for more positives. In a numerical example, they consider the case n = 18,918,900, and propose a design with 5460 pools which guarantees the identification of 2 positives, and can in addition identify up to 9 positives with 98.5% chance of success. By contrast, STD(n; 13; 13) contains 169 pools and guarantees the identification of 2 positives, and the identification of 9 positives is guaranteed with the 1369 pools of STD(n; 37; 37).

## Conclusion

In this paper, we have presented a new pooling design: the "shifted transversal design" (STD). We have proven that it constitutes an error-correcting solution to the pooling problem. This design is highly flexible: it can be tailored to deal efficiently with many experimental settings (i.e., numbers of variables, positives and errors). Finally, under a standard performance criterion, i.e. requiring that the correction of all errors and the identification of all variables' values be guaranteed mathematically, we have shown that STD compares favorably, in terms of numbers of pools, to the main previously described deterministic pooling designs.

This approach is being experimentally validated in collaboration with Marc Vidal's laboratory at the Dana Farber Cancer Institute, Boston. In a pilot project, pools have been generated with 940 AD-Y preys, using the STD(940;13;13) design, and we are screening the 169 resulting pools against 50 different baits. This experiment will provide estimations for the technical noise levels of their high-throughput 2-hybrid protocol, in addition to producing valuable interaction data and yielding a real-world evaluation of STD.

Although this work is motivated by protein interaction mapping, as we have been collaborating with Marc Vidal's group for several years, its scope is certainly not limited to high-throughput two-hybrid projects. Potential applications include a wide range of high-throughput PCR-based assays such as gene knockout projects, drug screening projects, and various proteomics studies. Furthermore, this general problem certainly has applications outside biology.

In practice, an important point is made in [20], where the author shows that his pooling design can be used to detect with high probability more positives than guaranteed. Simulations we have performed show that this observation is also true with STD: the gains can be increased substantially if one tolerates a small fraction of ambiguous variables that will need to be retested. However, these considerations are outside the scope of this paper, because we cannot study them analytically, but resort instead to computer simulations. Yet using such a strategy in practice with STD significantly improves the performance. For example, consider the case n = 10000 and t = 5, and suppose that the assay has an error-rate of 1%. To guarantee the identification of all variables' values, one must use 483 pools (with q = 23 and k = 21). However, if one tolerates up to 10 ambiguous variables, even when overestimating the error-rate to 2% for safety's sake, 143 pools prove amply sufficient. It is clear that this "ambiguity-tolerant" approach should be preferred in practical applications. This approach and the corresponding computer program, which performs simulations to select the STD parameter values best suited for a given application and includes original efficient algorithms for preparing the pools and decoding the outcomes, will be discussed in another paper.

Another interesting track will be to study the efficiency of pooling designs from the point of view of Shannon's information theory. We are planning to investigate STD's behavior in this context. Theorem 2 could prove useful for this.

Finally, the connection between STD and constructions based on superimposed codes, e.g. q-nary Reed-Solomon codes [11], warrants further studies.

## Methods

### Proof of theorem 1

Let i_1_,i_2 _∈ {0,...,n-1} with i_1 _≠ i_2_. Since each layer of pools is a partition of An
 MathType@MTEF@5@5@+=feaafiart1ev1aaatCvAUfKttLearuWrP9MDH5MBPbIqV92AaeXatLxBI9gBamrtHrhAL1wy0L2yHvtyaeHbnfgDOvwBHrxAJfwnaebbnrfifHhDYfgasaacH8akY=wiFfYdH8Gipec8Eeeu0xXdbba9frFj0=OqFfea0dXdd9vqai=hGuQ8kuc9pgc9s8qqaq=dirpe0xb9q8qiLsFr0=vr0=vr0dc8meaabaqaciaacaGaaeqabaWaaeGaeaaakeaaimaacqWFaeFqdaWgaaWcbaWexLMBbXgBcf2CPn2qVrwzqf2zLnharCGvLjhzH5wyaGqbaiaa+5gaaeqaaaaa@43C8@, there cannot be more than one pool per layer containing both A_i1 _and A_i2_. Furthermore, there exists a pool in layer *L*(*j*) that contains both A_i1 _and A_i2 _if and only if the columns for A_i1 _and A_i2 _are equal in M_j_, that is to say Cj,i1=Cj,i2
 MathType@MTEF@5@5@+=feaafiart1ev1aaatCvAUfKttLearuWrP9MDH5MBPbIqV92AaeXatLxBI9gBaebbnrfifHhDYfgasaacH8akY=wiFfYdH8Gipec8Eeeu0xXdbba9frFj0=OqFfea0dXdd9vqai=hGuQ8kuc9pgc9s8qqaq=dirpe0xb9q8qiLsFr0=vr0=vr0dc8meaabaqaciaacaGaaeqabaqabeGadaaakeaacqWGdbWqdaWgaaWcbaGaemOAaOMaeiilaWIaemyAaK2aaSbaaWqaaiabigdaXaqabaaaleqaaOGaeyypa0Jaem4qam0aaSbaaSqaaiabdQgaQjabcYcaSiabdMgaPnaaBaaameaacqaIYaGmaeqaaaWcbeaaaaa@39B4@. Therefore the number of pools of STD(n; q; q+1) that contain both i_1 _and i_2_, Card(*pools*_*q*+1_(*i*_1_) ∩ *pools*_*q*+1_(*i*_2_)), is the number of values of j in {0,...,q} such that Cj,i1=Cj,i2
 MathType@MTEF@5@5@+=feaafiart1ev1aaatCvAUfKttLearuWrP9MDH5MBPbIqV92AaeXatLxBI9gBaebbnrfifHhDYfgasaacH8akY=wiFfYdH8Gipec8Eeeu0xXdbba9frFj0=OqFfea0dXdd9vqai=hGuQ8kuc9pgc9s8qqaq=dirpe0xb9q8qiLsFr0=vr0=vr0dc8meaabaqaciaacaGaaeqabaqabeGadaaakeaacqWGdbWqdaWgaaWcbaGaemOAaOMaeiilaWIaemyAaK2aaSbaaWqaaiabigdaXaqabaaaleqaaOGaeyypa0Jaem4qam0aaSbaaSqaaiabdQgaQjabcYcaSiabdMgaPnaaBaaameaacqaIYaGmaeqaaaWcbeaaaaa@39B4@. However, the following equivalencies hold ∀ j ∈ {0,...,q-1} :

Cj,i1=Cj,i2⇔s(i1,j)≡s(i2,j)mod⁡q⇔∑c=0Γjc⋅⌊i1qc⌋≡∑c=0Γjc⋅⌊i2qc⌋mod⁡q⇔∑c=0Γjc⋅(⌊i1qc⌋−⌊i2qc⌋)≡0mod⁡q           (1)
 MathType@MTEF@5@5@+=feaafiart1ev1aaatCvAUfKttLearuWrP9MDH5MBPbIqV92AaeXatLxBI9gBaebbnrfifHhDYfgasaacH8akY=wiFfYdH8Gipec8Eeeu0xXdbba9frFj0=OqFfea0dXdd9vqai=hGuQ8kuc9pgc9s8qqaq=dirpe0xb9q8qiLsFr0=vr0=vr0dc8meaabaqaciaacaGaaeqabaqabeGadaaakqaaeeqaauaabeqabqaaaaqaaiabdoeadnaaBaaaleaacqWGQbGAcqGGSaalcqWGPbqAdaWgaaadbaGaeGymaedabeaaaSqabaGccqGH9aqpcqWGdbWqdaWgaaWcbaGaemOAaOMaeiilaWIaemyAaK2aaSbaaWqaaiabikdaYaqabaaaleqaaaGcbaGaeyi1HSnabaGaem4CamNaeiikaGIaemyAaK2aaSbaaSqaaiabigdaXaqabaGccqGGSaalcqWGQbGAcqGGPaqkcqGHHjIUcqWGZbWCcqGGOaakcqWGPbqAdaWgaaWcbaGaeGOmaidabeaakiabcYcaSiabdQgaQjabcMcaPaqaaiGbc2gaTjabc+gaVjabcsgaKnaaBaaaleaacqWGXbqCaeqaaaaaaOqaauaabeqacmaaaeGabaaibiaaxMaacqGHuhY2aeaadaaeWbqaaiabdQgaQnaaCaaaleqabaGaem4yamgaaaqaaiabdogaJjabg2da9iabicdaWaqaaiabfo5ahbqdcqGHris5aOGaeyyXIC9aayWaaeaadaWcaaqaaiabdMgaPnaaBaaaleaacqaIXaqmaeqaaaGcbaGaemyCae3aaWbaaSqabeaacqWGJbWyaaaaaaGccaGLWJVaay5+4dGaeyyyIO7aaabCaeaacqWGQbGAdaahaaWcbeqaaiabdogaJbaaaeaacqWGJbWycqGH9aqpcqaIWaamaeaacqqHtoWra0GaeyyeIuoakiabgwSixpaagmaabaWaaSaaaeaacqWGPbqAdaWgaaWcbaGaeGOmaidabeaaaOqaaiabdghaXnaaCaaaleqabaGaem4yamgaaaaaaOGaayj84laawUp+aaqaaiGbc2gaTjabc+gaVjabcsgaKnaaBaaaleaacqWGXbqCaeqaaaGcbiqaaypacaWLjaGaeyi1HSnabaWaaabCaeaacqWGQbGAdaahaaWcbeqaaiabdogaJbaaaeaacqWGJbWycqGH9aqpcqaIWaamaeaacqqHtoWra0GaeyyeIuoakiabgwSixpaabmaabaWaayWaaeaadaWcaaqaaiabdMgaPnaaBaaaleaacqaIXaqmaeqaaaGcbaGaemyCae3aaWbaaSqabeaacqWGJbWyaaaaaaGccaGLWJVaay5+4dGaeyOeI0YaayWaaeaadaWcaaqaaiabdMgaPnaaBaaaleaacqaIYaGmaeqaaaGcbaGaemyCae3aaWbaaSqabeaacqWGJbWyaaaaaaGccaGLWJVaay5+4daacaGLOaGaayzkaaGaeyyyIORaeGimaadabaGagiyBa0Maei4Ba8Maeiizaq2aaSbaaSqaaiabdghaXbqabaaaaOGaaCzcaiaaxMaadaqadaqaaiabigdaXaGaayjkaiaawMcaaaaaaa@BF67@

Since q is prime, Z/qZ is a field, namely the Galois field *GF*(*q*).

Furthermore, since i_1_≠i_2_, there exists at least one value c ∈ {0,...,Γ} such that (⌊i1qc⌋−⌊i2qc⌋)≠0
 MathType@MTEF@5@5@+=feaafiart1ev1aaatCvAUfKttLearuWrP9MDH5MBPbIqV92AaeXatLxBI9gBaebbnrfifHhDYfgasaacH8akY=wiFfYdH8Gipec8Eeeu0xXdbba9frFj0=OqFfea0dXdd9vqai=hGuQ8kuc9pgc9s8qqaq=dirpe0xb9q8qiLsFr0=vr0=vr0dc8meaabaqaciaacaGaaeqabaqabeGadaaakeaadaqadaqaamaagmaabaWaaSaaaeaacqWGPbqAdaWgaaWcbaGaeGymaedabeaaaOqaaiabdghaXnaaCaaaleqabaGaem4yamgaaaaaaOGaayj84laawUp+aiabgkHiTmaagmaabaWaaSaaaeaacqWGPbqAdaWgaaWcbaGaeGOmaidabeaaaOqaaiabdghaXnaaCaaaleqabaGaem4yamgaaaaaaOGaayj84laawUp+aaGaayjkaiaawMcaaiabgcMi5kabicdaWaaa@470D@ mod *q*. Indeed, i_1_,i_2 _∈ {0,...,n-1} and n ≤ q^Γ+1 ^entails that i1=∑c=0Γ(⌊i1qc⌋%q)⋅qc
 MathType@MTEF@5@5@+=feaafiart1ev1aaatCvAUfKttLearuWrP9MDH5MBPbIqV92AaeXatLxBI9gBaebbnrfifHhDYfgasaacH8akY=wiFfYdH8Gipec8Eeeu0xXdbba9frFj0=OqFfea0dXdd9vqai=hGuQ8kuc9pgc9s8qqaq=dirpe0xb9q8qiLsFr0=vr0=vr0dc8meaabaqaciaacaGaaeqabaqabeGadaaakeaacqWGPbqAdaWgaaWcbaGaeGymaedabeaakiabg2da9maaqahabaWaaeWaaeaadaGbdaqaamaalaaabaGaemyAaK2aaSbaaSqaaiabigdaXaqabaaakeaacqWGXbqCdaahaaWcbeqaaiabdogaJbaaaaaakiaawcp+caGL7JpacqGGLaqjcqWGXbqCaiaawIcacaGLPaaacqGHflY1cqWGXbqCdaahaaWcbeqaaiabdogaJbaaaeaacqWGJbWycqGH9aqpcqaIWaamaeaacqqHtoWra0GaeyyeIuoaaaa@4AA6@ and i2=∑c=0Γ(⌊i2qc⌋%q)⋅qc
 MathType@MTEF@5@5@+=feaafiart1ev1aaatCvAUfKttLearuWrP9MDH5MBPbIqV92AaeXatLxBI9gBaebbnrfifHhDYfgasaacH8akY=wiFfYdH8Gipec8Eeeu0xXdbba9frFj0=OqFfea0dXdd9vqai=hGuQ8kuc9pgc9s8qqaq=dirpe0xb9q8qiLsFr0=vr0=vr0dc8meaabaqaciaacaGaaeqabaqabeGadaaakeaacqWGPbqAdaWgaaWcbaGaeGOmaidabeaakiabg2da9maaqahabaWaaeWaaeaadaGbdaqaamaalaaabaGaemyAaK2aaSbaaSqaaiabikdaYaqabaaakeaacqWGXbqCdaahaaWcbeqaaiabdogaJbaaaaaakiaawcp+caGL7JpacqGGLaqjcqWGXbqCaiaawIcacaGLPaaacqGHflY1cqWGXbqCdaahaaWcbeqaaiabdogaJbaaaeaacqWGJbWycqGH9aqpcqaIWaamaeaacqqHtoWra0GaeyyeIuoaaaa@4AAA@, where % denotes the modulus (these are the unique decompositions of i_1 _and i_2 _in base q). Hence, i1−i2=∑c=0Γ((⌊i1qc⌋−⌊i2qc⌋)%q)⋅qc
 MathType@MTEF@5@5@+=feaafiart1ev1aaatCvAUfKttLearuWrP9MDH5MBPbIqV92AaeXatLxBI9gBaebbnrfifHhDYfgasaacH8akY=wiFfYdH8Gipec8Eeeu0xXdbba9frFj0=OqFfea0dXdd9vqai=hGuQ8kuc9pgc9s8qqaq=dirpe0xb9q8qiLsFr0=vr0=vr0dc8meaabaqaciaacaGaaeqabaqabeGadaaakeaacqWGPbqAdaWgaaWcbaGaeGymaedabeaakiabgkHiTiabdMgaPnaaBaaaleaacqaIYaGmaeqaaOGaeyypa0ZaaabCaeaadaqadaqaamaabmaabaWaayWaaeaadaWcaaqaaiabdMgaPnaaBaaaleaacqaIXaqmaeqaaaGcbaGaemyCae3aaWbaaSqabeaacqWGJbWyaaaaaaGccaGLWJVaay5+4dGaeyOeI0YaayWaaeaadaWcaaqaaiabdMgaPnaaBaaaleaacqaIYaGmaeqaaaGcbaGaemyCae3aaWbaaSqabeaacqWGJbWyaaaaaaGccaGLWJVaay5+4daacaGLOaGaayzkaaGaeiyjauIaemyCaehacaGLOaGaayzkaaaaleaacqWGJbWycqGH9aqpcqaIWaamaeaacqqHtoWra0GaeyyeIuoakiabgwSixlabdghaXnaaCaaaleqabaGaem4yamgaaaaa@5B3D@. Supposing that (⌊i1qc⌋−⌊i2qc⌋)≡0
 MathType@MTEF@5@5@+=feaafiart1ev1aaatCvAUfKttLearuWrP9MDH5MBPbIqV92AaeXatLxBI9gBaebbnrfifHhDYfgasaacH8akY=wiFfYdH8Gipec8Eeeu0xXdbba9frFj0=OqFfea0dXdd9vqai=hGuQ8kuc9pgc9s8qqaq=dirpe0xb9q8qiLsFr0=vr0=vr0dc8meaabaqaciaacaGaaeqabaqabeGadaaakeaadaqadaqaamaagmaabaWaaSaaaeaacqWGPbqAdaWgaaWcbaGaeGymaedabeaaaOqaaiabdghaXnaaCaaaleqabaGaem4yamgaaaaaaOGaayj84laawUp+aiabgkHiTmaagmaabaWaaSaaaeaacqWGPbqAdaWgaaWcbaGaeGOmaidabeaaaOqaaiabdghaXnaaCaaaleqabaGaem4yamgaaaaaaOGaayj84laawUp+aaGaayjkaiaawMcaaiabggMi6kabicdaWaaa@470F@ mod *q *for every c ∈ {0,...,Γ} would lead to i_1 _- i_2 _= 0, which is contradictory with the hypothesis that i_1_≠i_2_.

It follows that the above (1) can be seen as a non-zero polynomial (in j) of degree at most Γ on *GF*(*q*). As is well-known, such a polynomial has at most Γ roots in *GF*(*q*). That is to say, there are at most Γ values of j in {0,...,q-1} such that a pool of *L*(*j*) contains both A_i1_and A_i2_. This proves the theorem if Cq,i1≠Cq,i2
 MathType@MTEF@5@5@+=feaafiart1ev1aaatCvAUfKttLearuWrP9MDH5MBPbIqV92AaeXatLxBI9gBaebbnrfifHhDYfgasaacH8akY=wiFfYdH8Gipec8Eeeu0xXdbba9frFj0=OqFfea0dXdd9vqai=hGuQ8kuc9pgc9s8qqaq=dirpe0xb9q8qiLsFr0=vr0=vr0dc8meaabaqaciaacaGaaeqabaqabeGadaaakeaacqWGdbWqdaWgaaWcbaGaemyCaeNaeiilaWIaemyAaK2aaSbaaWqaaiabigdaXaqabaaaleqaaOGaeyiyIKRaem4qam0aaSbaaSqaaiabdghaXjabcYcaSiabdMgaPnaaBaaameaacqaIYaGmaeqaaaWcbeaaaaa@3A91@. Furthermore, if Cq,i1=Cq,i2
 MathType@MTEF@5@5@+=feaafiart1ev1aaatCvAUfKttLearuWrP9MDH5MBPbIqV92AaeXatLxBI9gBaebbnrfifHhDYfgasaacH8akY=wiFfYdH8Gipec8Eeeu0xXdbba9frFj0=OqFfea0dXdd9vqai=hGuQ8kuc9pgc9s8qqaq=dirpe0xb9q8qiLsFr0=vr0=vr0dc8meaabaqaciaacaGaaeqabaqabeGadaaakeaacqWGdbWqdaWgaaWcbaGaemyCaeNaeiilaWIaemyAaK2aaSbaaWqaaiabigdaXaqabaaaleqaaOGaeyypa0Jaem4qam0aaSbaaSqaaiabdghaXjabcYcaSiabdMgaPnaaBaaameaacqaIYaGmaeqaaaWcbeaaaaa@39D0@, the coefficient of j^Γ ^in (1) is zero by definition of *s*(*i*,*q*), and (1) is of degree at most Γ-1. Therefore if A_i1 _and A_i2 _are elements of the same pool in *L*(*q*), then there are at most Γ-1 pools in *L*(0),...,*L*(*q*-1) that contain both A_i1 _and A_i2_. This concludes the proof of theorem 1.

### Proof of theorem 2

Let j_1_,...,j_m _∈ {0,...,k-1} be the layer numbers and p_1_,...,p_m _∈ {0,...,q-1} be the pool indexes that define {P_1_,...,P_m_}: for every *h *∈ {1,...,m}, P_*h *_contains all variables of index i ∈ {0,...,n-1} such that *s*(*i*,*j*_*h*_) ≡ p_*h *_mod *q*.

|∩h=1mPh|
 MathType@MTEF@5@5@+=feaafiart1ev1aaatCvAUfKttLearuWrP9MDH5MBPbIqV92AaeXatLxBI9gBaebbnrfifHhDYfgasaacH8akY=wiFfYdH8Gipec8Eeeu0xXdbba9frFj0=OqFfea0dXdd9vqai=hGuQ8kuc9pgc9s8qqaq=dirpe0xb9q8qiLsFr0=vr0=vr0dc8meaabaqaciaacaGaaeqabaqabeGadaaakeaadaabdaqaamaauahabaGaemiuaa1aaSbaaSqaaiabdIgaObqabaaabaGaemiAaGMaeyypa0JaeGymaedabaGaemyBa0ganiablMIijbaakiaawEa7caGLiWoaaaa@38F4@ is the number of values i ∈ {0,...,n-1} such that: ∀ *h *∈ {1,...,m},

*s*(*i*,*j*_*h*_) ≡ p_*h *_mod *q*. Writing i=∑c=0Γαc⋅qc
 MathType@MTEF@5@5@+=feaafiart1ev1aaatCvAUfKttLearuWrP9MDH5MBPbIqV92AaeXatLxBI9gBaebbnrfifHhDYfgasaacH8akY=wiFfYdH8Gipec8Eeeu0xXdbba9frFj0=OqFfea0dXdd9vqai=hGuQ8kuc9pgc9s8qqaq=dirpe0xb9q8qiLsFr0=vr0=vr0dc8meaabaqaciaacaGaaeqabaqabeGadaaakeaacqWGPbqAcqGH9aqpdaaeWbqaaGGaciab=f7aHnaaBaaaleaaieGacqGFJbWyaeqaaaqaaiabdogaJjabg2da9iabicdaWaqaaiabfo5ahbqdcqGHris5aOGaeyyXICTaemyCae3aaWbaaSqabeaacqWGJbWyaaaaaa@3E51@ with *α*_0_,...,*α*_Γ _∈ {0,...,q-1} (this is the unique decomposition of i in base q), the above is equivalent to:

∀h∈{1,…,m},∑c=0Γαc⋅jhc≡phmod⁡q.     (2)
 MathType@MTEF@5@5@+=feaafiart1ev1aaatCvAUfKttLearuWrP9MDH5MBPbIqV92AaeXatLxBI9gBaebbnrfifHhDYfgasaacH8akY=wiFfYdH8Gipec8Eeeu0xXdbba9frFj0=OqFfea0dXdd9vqai=hGuQ8kuc9pgc9s8qqaq=dirpe0xb9q8qiLsFr0=vr0=vr0dc8meaabaqaciaacaGaaeqabaqabeGadaaakeaacqGHaiIicqWGObaAcqGHiiIZcqGG7bWEcqaIXaqmcqGGSaalcqWIMaYscqGGSaalcqWGTbqBcqGG9bqFcqGGSaaldaaeWbqaaGGaciab=f7aHnaaBaaaleaacqWGJbWyaeqaaaqaaiabdogaJjabg2da9iabicdaWaqaaiabfo5ahbqdcqGHris5aOGaeyyXICTaemOAaO2aa0baaSqaaiabdIgaObqaaiabdogaJbaakiabggMi6kabdchaWnaaBaaaleaacqWGObaAaeqaaOGagiyBa0Maei4Ba8Maeiizaq2aaSbaaSqaaiabdghaXbqabaGccqGGUaGlcaWLjaGaaCzcamaabmaabaGaeGOmaidacaGLOaGaayzkaaaaaa@5920@

This system can be written:

[1j1j12⋯j1Γ⋮⋮⋮⋮⋮1jmjm2⋯jmΓ]⋅[α0⋮αΓ]≡[p1⋮pm] mod⁡q.
 MathType@MTEF@5@5@+=feaafiart1ev1aaatCvAUfKttLearuWrP9MDH5MBPbIqV92AaeXatLxBI9gBaebbnrfifHhDYfgasaacH8akY=wiFfYdH8Gipec8Eeeu0xXdbba9frFj0=OqFfea0dXdd9vqai=hGuQ8kuc9pgc9s8qqaq=dirpe0xb9q8qiLsFr0=vr0=vr0dc8meaabaqaciaacaGaaeqabaqabeGadaaakeaadaWadaqaauaabeqaduaaaaqaaiabigdaXaqaaiabdQgaQnaaBaaaleaacqaIXaqmaeqaaaGcbaGaemOAaO2aa0baaSqaaiabigdaXaqaaiabikdaYaaaaOqaaiabl+UimbqaaiabdQgaQnaaDaaaleaacqaIXaqmaeaacqqHtoWraaaakeaacqWIUlstaeaacqWIUlstaeaacqWIUlstaeaacqWIUlstaeaacqWIUlstaeaacqaIXaqmaeaacqWGQbGAdaWgaaWcbaGaemyBa0gabeaaaOqaaiabdQgaQnaaDaaaleaacqWGTbqBaeaacqaIYaGmaaaakeaacqWIVlctaeaacqWGQbGAdaqhaaWcbaGaemyBa0gabaGaeu4KdCeaaaaaaOGaay5waiaaw2faaiabgwSixpaadmaabaqbaeqabmqaaaqaaGGaciab=f7aHnaaBaaaleaacqaIWaamaeqaaaGcbaGaeSO7I0eabaGae8xSde2aaSbaaSqaaiabfo5ahbqabaaaaaGccaGLBbGaayzxaaGaeyyyIO7aamWaaeaafaqabeWabaaabaGaemiCaa3aaSbaaSqaaiabigdaXaqabaaakeaacqWIUlstaeaacqWGWbaCdaWgaaWcbaGaemyBa0gabeaaaaaakiaawUfacaGLDbaacyGGTbqBcqGGVbWBcqGGKbazdaWgaaWcbaGaemyCaehabeaakiabc6caUaaa@7165@

**If m ≥ Γ+1: **consider the square sub-matrix composed of the first Γ+1 rows of the left member. Since P_1_,...,P_m _belong to different layers, the j_h _values are all distinct. Therefore, recalling that q is prime, this sub-matrix can be seen as a Vandermonde matrix with elements in the Galois field *GF*(*q*): it is nonsingular. This shows the existence of a unique tuple of values for *α*_0_,...,*α*_Γ _∈ {0,...,q-1} satisfying the first Γ+1 congruencies of (2). The remaining m-(Γ+1) congruencies may or may not be satisfied with these *α*_0_,...,*α*_Γ _values, and the corresponding i=∑c=0Γαc⋅qc
 MathType@MTEF@5@5@+=feaafiart1ev1aaatCvAUfKttLearuWrP9MDH5MBPbIqV92AaeXatLxBI9gBaebbnrfifHhDYfgasaacH8akY=wiFfYdH8Gipec8Eeeu0xXdbba9frFj0=OqFfea0dXdd9vqai=hGuQ8kuc9pgc9s8qqaq=dirpe0xb9q8qiLsFr0=vr0=vr0dc8meaabaqaciaacaGaaeqabaqabeGadaaakeaacqWGPbqAcqGH9aqpdaaeWbqaaGGaciab=f7aHnaaBaaaleaaieGacqGFJbWyaeqaaaqaaiabdogaJjabg2da9iabicdaWaqaaiabfo5ahbqdcqGHris5aOGaeyyXICTaemyCae3aaWbaaSqabeaacqWGJbWyaaaaaa@3E51@ might be too large (i.e. ≥ n); but in any case, there is at most one value of *i *satisfying the system: theorem 2 is proved when m ≥ Γ+1 (given that in this case *λ*_*m *_= 0).

**Otherwise, m ≤ Γ: **consider the square sub-matrix composed of the first m columns of the left member. Again, this sub-matrix is a Vandermonde matrix in *GF*(*q*), hence it is nonsingular. Consequently, given any values for *α*_m_,...,*α*_Γ_, there exists a unique tuple of values for *α*_0_,...,*α*_m-1 _in {0,...,q-1} satisfying (2) (simply shift the terms in *α*_m_,...,*α*_Γ _to the right member). The question therefore becomes: how many tuples of values for *α*_m_,...,*α*_Γ _exist, such that i=∑c=0Γαc⋅qc<n
 MathType@MTEF@5@5@+=feaafiart1ev1aaatCvAUfKttLearuWrP9MDH5MBPbIqV92AaeXatLxBI9gBaebbnrfifHhDYfgasaacH8akY=wiFfYdH8Gipec8Eeeu0xXdbba9frFj0=OqFfea0dXdd9vqai=hGuQ8kuc9pgc9s8qqaq=dirpe0xb9q8qiLsFr0=vr0=vr0dc8meaabaqaciaacaGaaeqabaqabeGadaaakeaacqqGPbqAcqGH9aqpdaaeWbqaaGGaciab=f7aHnaaBaaaleaacqWGJbWyaeqaaaqaaiabdogaJjabg2da9iabicdaWaqaaiabfo5ahbqdcqGHris5aOGaeyyXICTaemyCae3aaWbaaSqabeaacqWGJbWyaaGccqGH8aapcqqGUbGBaaa@40BA@ where *α*_0_,...,*α*_m-1 _are determined by *α*_m_,...,*α*_Γ _as explained above. To answer this, consider the unique decomposition of n-1 in base q: n−1=∑c=0Γβc⋅qc
 MathType@MTEF@5@5@+=feaafiart1ev1aaatCvAUfKttLearuWrP9MDH5MBPbIqV92AaeXatLxBI9gBaebbnrfifHhDYfgasaacH8akY=wiFfYdH8Gipec8Eeeu0xXdbba9frFj0=OqFfea0dXdd9vqai=hGuQ8kuc9pgc9s8qqaq=dirpe0xb9q8qiLsFr0=vr0=vr0dc8meaabaqaciaacaGaaeqabaqabeGadaaakeaacqWGUbGBcqGHsislcqaIXaqmcqGH9aqpdaaeWbqaaGGaciab=j7aInaaBaaaleaacqWGJbWyaeqaaaqaaiabdogaJjabg2da9iabicdaWaqaaiabfo5ahbqdcqGHris5aOGaeyyXICTaemyCae3aaWbaaSqabeaacqWGJbWyaaaaaa@4034@, where βc=⌊n−1qc⌋%q
 MathType@MTEF@5@5@+=feaafiart1ev1aaatCvAUfKttLearuWrP9MDH5MBPbIqV92AaeXatLxBI9gBaebbnrfifHhDYfgasaacH8akY=wiFfYdH8Gipec8Eeeu0xXdbba9frFj0=OqFfea0dXdd9vqai=hGuQ8kuc9pgc9s8qqaq=dirpe0xb9q8qiLsFr0=vr0=vr0dc8meaabaqaciaacaGaaeqabaqabeGadaaakeaaiiGacqWFYoGydaWgaaWcbaGaem4yamgabeaakiabg2da9maagmaabaWaaSaaaeaacqWGUbGBcqGHsislcqaIXaqmaeaacqWGXbqCdaahaaWcbeqaaiabdogaJbaaaaaakiaawcp+caGL7JpacqGGLaqjcqWGXbqCaaa@3E77@ for c ∈ {0,...,Γ}. Under this representation, it is clear that i < n, i.e. i ≤ n-1, if and only if:

*α*_Γ _< *β*_Γ _or

 (αΓ=βΓ and(αΓ<βΓ or(... and (αm<βm or(αm=βm and ∑c=0Γac⋅qc<n))...))).
 MathType@MTEF@5@5@+=feaafiart1ev1aaatCvAUfKttLearuWrP9MDH5MBPbIqV92AaeXatLxBI9gBaebbnrfifHhDYfgasaacH8akY=wiFfYdH8Gipec8Eeeu0xXdj9arFj0xirFj0dXdbba91qpepeI8k8fiI+fsY=rqGqpepae9pg0db9vqaiVgFr0xfr=xfr=xc9adbaqaaeGaciGaaiaabeqaaeqabiWaaaGcbaGaeeiiaasbaeaabmGaaaqaaiabcIcaOiabeg7aHnaaBaaaleaacqqHtoWraeqaaOGaeyypa0JaeqOSdi2aaSbaaSqaaiabfo5ahbqabaGccqqGGaaicqqGHbqycqqGUbGBcqqGKbazaeaacqqGOaakcqaHXoqydaWgaaWcbaGaeu4KdCeabeaakiabgYda8iabek7aInaaBaaaleaacqqHtoWraeqaaOGaeeiiaaIaee4Ba8MaeeOCaihabaaabaGaeiikaGIaeeOla4IaeeOla4IaeeOla4IaeeiiaaIaeeyyaeMaeeOBa4MaeeizaqMaeeiiaaIaeeikaGIaeqySde2aaSbaaSqaaiabb2gaTbqabaGccqGH8aapcqaHYoGydaWgaaWcbaGaeeyBa0gabeaakiabbccaGiabb+gaVjabbkhaYbqaaaqaaiabbIcaOiabeg7aHnaaBaaaleaacqqGTbqBaeqaaOGaeyypa0JaeqOSdi2aaSbaaSqaaiabb2gaTbqabaGccqqGGaaicqqGHbqycqqGUbGBcqqGKbazcqqGGaaidaaeWbqaaiabbggaHnaaBaaaleaacqqGJbWyaeqaaaqaaiabbogaJjabb2da9iabbcdaWaqaaiabfo5ahbqdcqGHris5aOGaeyyXICTaeeyCae3aaWbaaSqabeaacqqGJbWyaaGccqGH8aapcqqGUbGBcqqGPaqkcqqGPaqkcqqGUaGlcqqGUaGlcqqGUaGlcqqGPaqkcqqGPaqkcqqGPaqkcqqGUaGlaaaaaa@7E53@

For each c ∈ {m,...,Γ}, the branch ending at (*α*_c _<*β*_c_) yields *β*_c_·q^(c-m) ^different tuples. Indeed, for d > c *α*_d _= *β*_d _in this branch, and *α*_0_,...,*α*_m-1 _are bound to *α*_m_,...,*α*_Γ_: there are *β*_c _possible choices for *α*_c_, and q choices each for *α*_m_,...,*α*_c-1_. As to the final branch, it can yield at most one solution, given that all the *α *values are set or bound in this branch.

Consequently, there are a total of λm=∑c=mΓβc⋅qc−m
 MathType@MTEF@5@5@+=feaafiart1ev1aaatCvAUfKttLearuWrP9MDH5MBPbIqV92AaeXatLxBI9gBaebbnrfifHhDYfgasaacH8akY=wiFfYdH8Gipec8Eeeu0xXdbba9frFj0=OqFfea0dXdd9vqai=hGuQ8kuc9pgc9s8qqaq=dirpe0xb9q8qiLsFr0=vr0=vr0dc8meaabaqaciaacaGaaeqabaqabeGadaaakeaaiiGacqWF7oaBdaWgaaWcbaGaemyBa0gabeaakiabg2da9maaqahabaGae8NSdi2aaSbaaSqaaiabdogaJbqabaaabaGaem4yamMaeyypa0JaemyBa0gabaGaeu4KdCeaniabggHiLdGccqGHflY1cqWGXbqCdaahaaWcbeqaaiabdogaJjabgkHiTiabd2gaTbaaaaa@42FF@ or *λ*_*m*_+1 solutions: theorem 2 is also proved when m ≤ Γ.

## Figures and Tables

**Table 1:** Choosing the optimal value for the number of pools per layer, q

**q**	**Γ**	**k**	**v**	**gain**
≤ 13	≥ 3	≥ 16	k > q+1, can't use these values	
17	3	16	272	36.8
19	3	16	304	32.9
23	2	11	253	39.5
29	2	11	319	31.3
...	2	11	...	...
97	2	11	1067	9.4
101	1	6	606	16.5

This table shows the gains obtained with various q values, when the total number of variables to be tested is n = 10000 and the number of expected positives is t = 5, in a noiseless experiment (E = 0). Γ is the compression power (i.e. logarithm of n in base q, see Preliminaries in Results(1) section), k is the number of layers, v is the number of pools (i.e. k·q), and the gain is defined as n/v. By construction, STD requires k ≤ q+1; and to guarantee the identification of t positives while correcting E errors, section 3.3 showed that we must choose k = t·Γ+2·E+1; in this example, k = 5Γ+1. Often, the smallest useable q (i.e., satisfying k ≤ q+1), q_min_, yields the highest gain, but this is not always the case. In this example, q_min _= 17, but q = 23 (smallest q such that Γ = 2) yields the highest gain: 39.5.

**Table 2:** Gains obtained when the identification of 3 positives and the correction of 2 errors is guaranteed (t = 3, E = 2)

**n**	**q_opt_**	**pool size**	**k**	**v**	**gain**
100	11	9	8	88	1.1
1000	11	91	11	121	8.3
10^4^	13	769	14	182	55
10^5^	19	5263	14	266	376
10^6^	19	52631	17	323	3096

For each value of n (total number of variables), the optimal q value q_opt _has been calculated, as well as the associated pool size, the number of layers k, the total number of pools v, and the gain.

## References

[B1] Reboul J, Vaglio P, Rual JF, Lamesch P, Martinez M, Armstrong CM, Li S, Jacotot L, Bertin N, Janky R, Moore T, Hudson JR Jr, Hartley JL, Brasch MA, Vandenhaute J, Boulton S, Endress GA, Jenna S, Chevet E, Papasotiropoulos V, Tolias PP, Ptacek J, Snyder M, Huang R, Chance MR, Lee H, Doucette-Stamm L, Hill DE, Vidal M (2003). *C. elegans *ORFeome version 1.1: experimental verification of the genome annotation and resource for proteome-scale protein expression. Nat Genet.

[B2] Walhout A, Sordella R, Lu X, Hartley J, Temple GF, Brasch MA, Thierry-Mieg N, Vidal M (2000). Protein interaction mapping in *C. elegans *using proteins involved in vulval development. Science.

[B3] Davy A, Bello P, Thierry-Mieg N, Vaglio P, Hitti J, Doucette-Stamm L, Thierry-Mieg D, Reboul J, Boulton S, Walhout AJ, Coux O, Vidal M (2001). A protein-protein interaction map of the *Caenorhabditis elegans *26S proteasome. EMBO Rep.

[B4] Evans G, Lewis K (1989). Physical mapping of complex genomes by cosmid multiplex analysis. Proc Natl Acad Sci USA.

[B5] Zwaal R, Broeks A, van Meurs J, Groenen J, Plasterk RH (1993). Target-selected gene inactivation in *Caenorhabditis elegans *by using a frozen transposon insertion mutant bank. Proc Natl Acad Sci USA.

[B6] Cai W, Chen R, Gibbs R, Bradley A (2001). A clone-array pooled shotgun strategy for sequencing large genomes. Genome Res.

[B7] Balding D, Bruno W, Knill E, Torney D (1996). A comparative survey of non-adaptive pooling designs. Genetic mapping and DNA sequencing.

[B8] Bruno W, Knill E, Balding D, Bruce D, Doggett NA, Sawhill WW, Stallings RL, Whittaker CC, Torney DC (1995). Efficient pooling designs for library screening. Genomics.

[B9] Ngo H, Du DZ (2000). A survey on combinatorial group testing algorithms with applications to DNA library screening. DIMACS Ser Discrete Math Theoret Comput Sci.

[B10] Du DZ, Hwang F (2000). Combinatorial Group Testing and Its Applications, 2^nd ^edn.

[B11] Kautz W, Singleton H (1964). Nonrandom binary superimposed codes. IEEE Trans Inform Theory.

[B12] Balding D, Torney D (1996). Optimal pooling designs with error correction. J Comb Theory Ser A.

[B13] Barillot E, Lacroix B, Cohen D (1991). Theoretical analysis of library screening using a N-dimensional pooling strategy. Nucl Acids Res.

[B14] Chateauneuf M, Colbourn C, Kreher D, Lamken E, Torney D (1999). Pooling, lattice square, and union jack designs. Ann Comb.

[B15] Macula A (1996). A simple construction of d-disjunct matrices with certain constant weights. Discrete Math.

[B16] Ngo H, Du DZ (2002). New constructions of non-adaptive and error-tolerance pooling designs. Discrete Math.

[B17] Reed I, Solomon G (1960). Polynomial codes over certain finite fields. J Soc Ind Appl Math.

[B18] Balding D, Torney D (1997). The design of pooling experiments for screening a clone map. Fungal genet, biol.

[B19] Colbourn C, Mathon R, Colbourn C, Dinitz J (1996). Steiner systems. The CRC Handbook of Combinatorial Designs.

[B20] Macula A (1999). Probabilistic nonadaptive group testing in the presence of errors and dna library screening. Ann Comb.

